# Ultraviolet radiation as a double-edged regulator of melanocyte function in vitiligo therapy and melanoma carcinogenesis

**DOI:** 10.3389/fmed.2026.1823921

**Published:** 2026-06-01

**Authors:** Heng-Heng Fan, Yan-Yan Chen, Yun Geng, Yi-Fei Yang, Hui Xu, Yu-Yun Xiong, Yun-Wen Zheng, Yu-Mei Li

**Affiliations:** 1 Department of Dermatology, Institute of Regenerative Medicine, Affiliated Hospital of Jiangsu University, Jiangsu University, Zhenjiang, Jiangsu, China; 2 Guangdong Provincial Key Laboratory of Large Animal Models for Biomedicine, South China Institute of Large Animal Models for Biomedicine, Wuyi University, Jiangmen, Guangdong, China; 3 School of Pharmacy and Food Engineering, Wuyi University, Jiangmen, Guangdong, China; 4 Department of Medical and Life Sciences, Faculty of Pharmaceutical Sciences, Tokyo University of Science, Chiba, Japan; 5 Center for Stem Cell Biology and Regenerative Medicine, Institute of Medical Science, The University of Tokyo, Tokyo, Japan

**Keywords:** melanocyte, melanoma, phototherapy, ultraviolet, vitiligo

## Abstract

Vitiligo is a chronic autoimmune disorder characterized by selective melanocyte loss and progressive depigmentation. Narrowband ultraviolet B (NB-UVB) and excimer-based phototherapy are widely regarded as standard treatments in clinical practice and remain the most effective approaches for inducing repigmentation. However, excessive or uncontrolled ultraviolet radiation (UVR) exposure can impair skin barrier function, cause melanocyte dysfunction, and increase the risk of photoaging and carcinogenesis. This apparent paradox arises from the bidirectional biological effects of UVR: on the one hand, UVR activates melanocytes and promotes skin pigmentation through coordinated effects on melanocyte maturation, proliferation, and melanin synthesis, while on the other hand, UVR can induce oxidative stress, DNA damage, apoptosis, and genomic instability. Therefore, combination strategies incorporating JAK inhibitors, platelet-rich plasma, or cellular grafting techniques have been increasingly explored in clinical practice. In this review, we provide an integrated perspective on the dual effects of UVR on melanocyte biology, discuss emerging combination therapies for vitiligo, and highlight the mechanistic links between phototherapy, melanocyte homeostasis, and melanoma risk.

## 1 Introduction

Melanocytes are specialized pigment-producing cells located in the epidermis that synthesize melanin and transfer it to neighboring keratinocytes via dendritic processes (1). In vertebrates, the melanocyte lineage originates from embryonic neural crest cells (2). During embryogenesis, neural crest cells delaminate from the dorsal neural tube, migrate to multiple target tissues, and differentiate into diverse cell types, including melanoblasts. These melanoblasts subsequently migrate to the basal layer of the epidermis, where they undergo terminal differentiation to form functional melanocytes. In parallel, a distinct population of melanoblasts migrates into developing hair follicles. Within this niche, they give rise to differentiated melanocytes in the hair follicle bulb and to melanocyte stem cells residing in the bulge and hair germ regions, which serve as a reservoir for melanocyte regeneration during hair cycling and skin repair.

The primary biological function of melanin is to protect the skin against ultraviolet radiation (UVR)–induced damage. The ultraviolet (UV) spectrum (100–400 nm) is conventionally divided into three wavelength bands: UVA (315–400 nm), UVB (280–315 nm), and UVC (100–280 nm) (3). Since UVC is effectively absorbed by the stratospheric ozone layer, only UVA (approximately 90–99%) and UVB (approximately 1–10%) reach the Earth’s surface, making them the principal contributors to UV-associated skin pathology (4). As summarized in Table 1, the depth of UVR penetration is wavelength dependent: UVA penetrates deeply into the dermis (up to ~1,000 μm), whereas UVB is largely absorbed within the epidermis and superficial dermis (~160–180 μm). Exposure to UVR elicits diverse biological responses in melanocytes (5). Low-dose UV exposure (<0.06 J/cm^2^) enhances epidermal pigmentation and increases melanin content, thereby contributing to photoprotection (6). In contrast, high-dose or chronic UV exposure (≥0.06 J/cm^2^) promotes melanocyte senescence, genomic instability, tumorigenesis, and cell death (7). This review highlights the bidirectional effects of UVR on melanocyte function, with particular emphasis on balancing its therapeutic relevance in vitiligo against its potential photobiological and carcinogenic risks. We propose that UVR modulates melanocyte biology through three interrelated mechanisms: (1) stimulation of melanocyte proliferation and melanogenesis under controlled exposure; (2) reshaping of the local immune and oxidative microenvironment; and (3) induction of genomic stress and oncogenic susceptibility under excessive or prolonged exposure. While these biological effects underpin the clinical efficacy of UV-based therapies in vitiligo, they also underscore the importance of carefully balancing therapeutic benefit against long-term carcinogenic risk.

**Table 1:** Classification and impact of UVR.

UVR	Wavelength	Depth of skin	Cells	Effect	Disease	Treatment of pathogenesis
UVA	315–400 nm (3)	Dermis (1,000 μm)	Keratinocytes (basal epidermis) Fibroblasts (dermis) Dendritic cells, endothelial cells, immune cells	Deep penetration (~1,000 μm) Oxidative stress, DNA damage Photoaging, immunosuppression	Melanoma Photoaging (wrinkles) Vitiligo (oxidative stress)	Antioxidants (prevention) PUVA (psoriasis/vitiligo)
UVB	280–315 nm (3)	Epidermis or upper dermis (160-180 μm)	Epidermal keratinocytes Melanocytes Langerhans cells	Erythema (sunburn) DNA damage (thymine dimers) Vitamin D synthesis	Non-melanoma skin cancer (BCC, SCC) Psoriasis Vitiligo	NB-UVB (311 nm) for vitiligo/psoriasis Excimer laser (308 nm)
UVC	100–280 nm (3)	-	Superficial skin cells (mostly blocked by ozone)	Germicidal (destroys pathogens) Severe DNA damage	Rare (artificial exposure risks)	–

**Figure 1:**
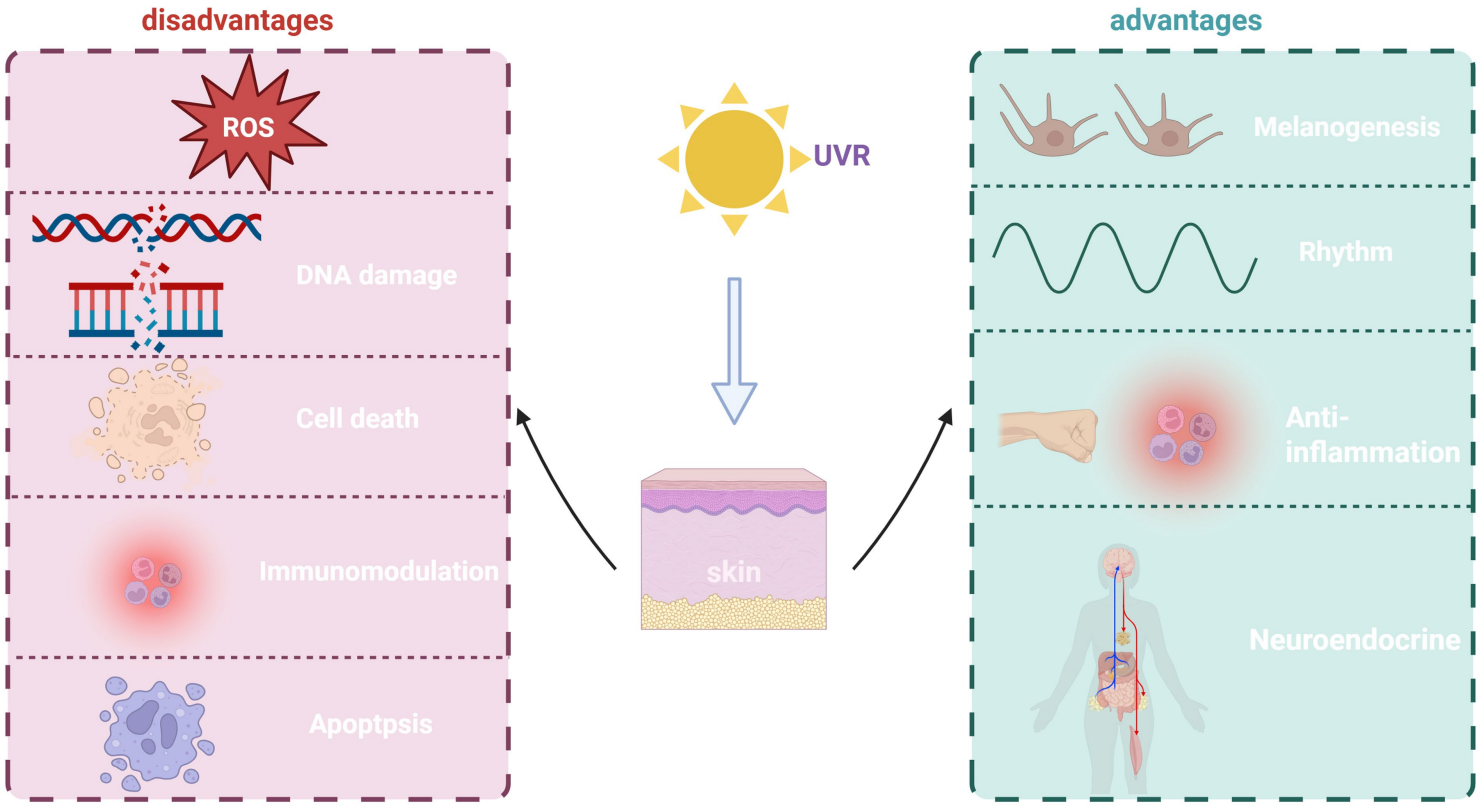
Effects of UVR on the skin (created at https://BioRender.com).

From an evolutionary perspective, UVR has also been proposed to have contributed to prebiotic chemical reactions on early Earth by providing free energy for photochemical processes relevant to the emergence of biological molecules. Although there is no direct evidence that such pre-cellular processes are preserved as discrete mechanisms in vitiligo or melanoma, several fundamental photochemical principles remain biologically relevant in modern melanocyte biology. These include chromophore-mediated photon absorption, redox reactions, ROS/RNS generation, DNA photoproduct formation, and stress-response signaling. In this context, melanin may be viewed not only as a UV-protective pigment but also as a biologically active chromophore that converts UV energy into chemical and cellular signals. Dysregulation of these photochemical processes may contribute to melanocyte damage in vitiligo and, under chronic or excessive UV exposure, to genomic instability and melanoma progression. Therefore, this evolutionary perspective on UV-driven photochemistry provides a conceptual background for understanding why UV exposure can act as both a regulator of melanocyte homeostasis and a driver of melanocyte pathology (8, 9) (Figure 1).

## 2 Beneficial effects of UVR on melanocyte function

UVR elicits adaptive responses in melanocytes that are essential for pigmentation, environmental sensing, and overall skin homeostasis. Accumulating evidence indicates that UVR exposure promotes melanin production in melanocytes through both direct and indirect mechanisms, thereby mitigating the cytotoxic effects of UV stress. This section delineates the molecular and cellular pathways by which UVR supports melanocyte function under physiological and pathological conditions.

### 2.1 Melanogenesis

UVR rapidly activates melanocytes and promotes skin pigmentation through coordinated effects on melanocyte maturation, proliferation, and melanin synthesis. Microphthalmia-associated transcription factor (MITF) serves as the key regulator of melanocyte development, function, and survival. UVB induces the expression of melanogenesis-related genes, including *MITF*, *tyrosinase (TYR), and tyrosinase-related protein 1 (TYRP1)*, thereby initiating melanogenic programs in melanocytes (10).

Recent studies have employed forskolin to activate cyclic adenosine monophosphate (cAMP) signaling as a surrogate for UVR stimulation. Single-cell analyses revealed that melanocytes dynamically transition between distinct functional states in response to cAMP activation: repeated stimulation preferentially expands a proliferative state, whereas intermittent exposure enriches a highly pigmented state. These state transitions are tightly linked to dynamic, dose-dependent regulation of MITF expression, underscoring its role as a molecular rheostat governing melanocyte fate decision (11). Beyond transcriptional regulation, UVB exposure promotes melanogenesis by modulating intracellular calcium signaling and melanosomal pH. In melanocytes, UVB activates transient receptor potential ankyrin 1 (TRPA1) channels, triggering calcium influx and subsequent alkalinization of melanosomes. These changes enhance *tyrosinase* (*TYR*) activity, thereby facilitating melanin synthesis (12). In addition to its direct effects on differentiated melanocytes, UVR also acts on melanocyte precursor cells. UVR exposure activates the KIT receptor in melanocyte precursors, stimulating their proliferation and differentiation into mature, TRP1-positive pigmented melanocytes (13).

Furthermore, UVR exposure modulates melanogenesis through complex interactions with neighboring cell types, particularly keratinocytes. The crosstalk between keratinocytes and melanocytes represents a central regulatory axis in cutaneous pigmentation. Upon UV exposure, keratinocytes secrete multiple paracrine mediators that regulate melanocyte activity. Among these, *α*-melanocyte-stimulating hormone (α-MSH) binds to melanocortin 1 receptor (MC1R) on melanocytes and activates downstream melanogenic signaling, thereby upregulating the expression of melanogenic genes, including *TYR, TYRP1*, and dopachrome tautomerase (*DCT*) (1, 12, 14, 15). In contrast, interleukin-1β (IL-1β) acts through its cognate IL-1 receptor and contributes to UVR-induced inflammatory and paracrine signaling within the epidermal microenvironment, which may indirectly influence melanocyte function. In addition to soluble cytokines and hormones, UVB radiation induces keratinocytes to release exosome-associated microRNAs, such as miR-365b-5p, which further modulate melanin synthesis in melanocytes (16). Moreover, UVB exposure activates the cutaneous sympathetic nervous system, leading to increased norepinephrine release. This, in turn, upregulates *β*2-adrenergic receptor (β2-AR) expression in keratinocytes and indirectly influences melanogenesis in melanocytes through the activation of the AP-1 signaling pathway.

UVR-induced melanogenesis is mediated by multiple signaling pathways. Both UVA and UVB enhance the phosphorylation of ERK1/2 and MITF activation, reinforcing transcriptional control of melanogenic enzymes (17). The cAMP/protein kinase A (PKA)/cAMP response element-binding protein (CREB) signaling axis pathway is the main pathway for melanogenesis after UVR exposure. This process involves the extracellular conversion of ATP to adenosine by CD39 and CD73, followed by adenosine binding to A_2A_ and A_2B_ receptors, which further amplifies intracellular cAMP signaling and melanin synthesis. In addition to classical second messenger pathways, UVR promotes melanogenesis by modulating mitochondrial dynamics. Enhanced mitochondrial fusion has been shown to activate ERK1/2 signaling, leading to increased MITF expression and upregulation of downstream melanogenic enzymes (18). Moreover, UVR regulates melanocyte pigmentation through activation of melanopsin (OPN4) and calcium-dependent signaling pathways, including CaMKII, nitric oxide, and cyclic guanosine monophosphate (cGMP) (19). Meanwhile, activation of the Wnt/β-catenin pathway by UV exposure also contributes to melanocyte proliferation, differentiation, and sustained melanin production (14, 20).

Importantly, melanogenic signaling is subject to intrinsic negative regulation. *α*-MSH activates downstream MITF signaling, resulting in upregulation of NOX4 expression. Activation of the MITF–NOX4 axis enhances reactive oxygen species (ROS) production, which in turn negatively regulates melanin synthesis (21). This MITF–NOX4 axis represents a context-dependent feedback mechanism that fine-tunes melanocyte redox homeostasis and prevents excessive melanogenesis under sustained stimulation.

### 2.2 UV-related eumelanogenesis and pheomelanogenesis

UV-induced melanogenesis should be interpreted not only as an increase in total melanin content but also as a wavelength- and MC1R-dependent shift in melanin quality. In human melanocytes, activation of the POMC/α-MSH/MC1R/cAMP axis preferentially promotes eumelanogenesis, resulting in the production of brown-black eumelanin. Eumelanin has broad UV-absorbing and antioxidant properties, thereby reducing UV-induced ROS accumulation, CPD formation, and mutagenesis. In contrast, impaired MC1R signaling, which is common in red-hair/fair-skin phenotypes, favors pheomelanogenesis and the production of red-yellow sulfur-containing pheomelanin. Pheomelanin is less efficient at UV shielding and can promote oxidative stress through photoreactive and pro-oxidant reactions, thereby increasing melanocyte vulnerability even when total pigmentation is present (22–24).

This distinction is relevant to both vitiligo and melanoma. In vitiligo, reduced melanocyte number and impaired melanogenic capacity decrease the photoprotective reservoir of eumelanin, while oxidative stress may further disrupt melanogenic enzymes and melanosomal function. Thus, controlled NB-UVB can be beneficial by stimulating melanocyte regeneration and eumelanin-biased pigmentation, whereas excessive UVR may aggravate oxidative injury in melanocytes with insufficient antioxidant resilience. In melanoma biology, the eumelanin/pheomelanin balance helps explain why pigmentation is not uniformly protective: eumelanin-rich pigmentation generally reduces UV-induced DNA damage, whereas pheomelanin-rich phenotypes are associated with increased melanoma susceptibility through both UV-dependent and UV-independent oxidative mechanisms. Therefore, future clinical interpretation of UV responses in vitiligo should consider not only repigmentation rate but also pigment quality, MC1R status, and oxidative stress burden (25).

### 2.3 Circadian regulation

Melanogenesis is the most important function of melanocytes, but recently, other functions of melanocytes have also attracted people’s attention. Circadian rhythms are highly conserved biological processes that regulate a wide range of physiological functions across species. Light represents the primary environmental input to the circadian system and functions as a dominant *zeitgeber*. In ectotherms, through opsin-mediated photoreception in light-sensing neurons of the eye and central nervous system, light regulates the circadian synthesis and secretion of melatonin. This photic regulation of melatonin signaling, in turn, influences pigment distribution, cellular proliferation, and tissue homeostasis (26).

Opsin-mediated photoreception, particularly melanopsin (OPN4), plays an important role in regulating pigmentation in mammals following UVR exposure (19). Both *BMAL1* and *PER1* are core clock genes involved in circadian regulation. In melanocytes, UVA exposure has been reported to increase melanin content, at least in part, through OPN4-dependent suppression of PER1 expression. In contrast, OPN4-deficient (Opn4^KO) melanocytes fail to exhibit UVA-induced PER1 suppression and show impaired pigmentation responses, underscoring the essential role of OPN4 in linking light sensing, circadian signaling, and melanogenesis (27). Clinically, OPN4 expression declines with melanoma progression and is inversely correlated with BMAL1 expression, while higher BMAL1 levels are associated with improved patient survival (28). In addition, UVR may alter melanocyte-associated neuroendocrine rhythms by suppressing nocturnal melatonin secretion while elevating daytime serotonin levels (29). Although current evidence linking UVR-induced circadian disruption predominantly derives from melanoma models, these findings raise the possibility that dysregulation of melanocyte circadian rhythms may contribute to melanocyte transformation and carcinogenesis, warranting further investigation.

UVR markedly influences melanocyte-associated endocrine activity through both direct and indirect mechanisms. UV exposure upregulates cutaneous expression of corticotropin-releasing hormone (CRH) and pro-opiomelanocortin (POMC), the precursor of *α*-melanocyte-stimulating hormone (α-MSH) (30). Binding of α-MSH to the melanocortin-1 receptor (MC1R) activates adenylate cyclase, leading to increased intracellular cyclic adenosine monophosphate (cAMP) levels (31). This signaling cascade results in phosphorylation of cAMP response element-binding protein (CREB), thereby promoting eumelanin synthesis and enhancing photoprotection against UV-induced DNA damage (32).

UVR also regulates melanocyte function through activation of the nuclear factor erythroid 2–related factor 2 (NRF2) signaling pathway, inducing antioxidant enzymes, such as heme oxygenase-1, to mitigate oxidative stress (33). In parallel, melanocortin-1 receptor (MC1R) polymorphisms can impair α-melanocyte-stimulating hormone (*α*-MSH) signaling, leading to reduced melanogenic capacity and increased UV sensitivity. Conversely, UVB exposure enhances MC1R expression and functional responsiveness, thereby further potentiating α-MSH-mediated pigmentation (30).

In addition, melanotrophs in the pituitary gland express the short-wavelength-sensitive opsin, Opn5m, which mediates intracellular calcium influx in response to UV exposure (31). This calcium-dependent signaling promotes the release of melanocyte-stimulating hormone (MSH), leading to upregulation of tyrosinase expression and stimulation of melanogenesis. Genetic ablation of *Opn5m* reduces tyrosinase activity and results in hypopigmentation, confirming its essential role in UV-responsive endocrine regulation of pigmentation (31).

Collectively, UVR coordinates melanocyte endocrine regulation through CRH/POMC/α-MSH signaling, NRF2-mediated antioxidant responses, MC1R modulation, and pituitary-derived, Opn5m-dependent MSH release (30). Together, these interconnected pathways act in concert to enhance melanin production and preserve cutaneous homeostasis in response to UV-induced stress.

### 2.4 Melanocytes as UV-sensing neuroendocrine regulators

Beyond their classical role as pigment-producing cells, melanocytes can be viewed as UV-sensing regulatory cells that translate physical radiation into chemical, paracrine, and neuroendocrine signals. This concept, emphasized in the work of Slominski, Paus, and colleagues, is particularly relevant because melanocytes are neural crest-derived cells equipped with photoreceptive molecules, melanogenic enzymes, stress-response pathways, and multiple neuroendocrine mediators. After UV exposure, melanocytes and neighboring keratinocytes activate local CRH/POMC/ACTH/α-MSH signaling, release cytokines and growth factors, and modulate redox and nitric oxide pathways. Through melanosome transfer, dendritic communication, soluble mediators, and extracellular vesicles, melanocytes can influence keratinocytes, immune cells, fibroblasts, endothelial cells, and hair follicle melanocyte stem-cell niches (25, 30).

Within this framework, melanin itself should be considered more than a passive UV screen. As a broadband chromophore, melanin absorbs UV photons and dissipates part of the absorbed energy, thereby protecting nuclear DNA in melanocytes and surrounding keratinocytes. However, melanin and its intermediates can also participate in electron-transfer reactions, chemiexcitation, and ROS/RNS generation, particularly after UVA exposure. This chromophore-like, signal-transducing property of melanin helps bridge the beneficial and detrimental sides of UV biology: in vitiligo, insufficient or unstable melanocyte–melanin units reduce tissue photoprotection and may amplify oxidative autoimmunity; in melanoma, persistent UV-driven melanogenic and oxidative signaling can create a microenvironment permissive for mutagenesis, immune escape, and tumor progression (34).

## 3 Detrimental effects of excessive UVR

### 3.1 Oxidative stress

UVR induces the generation of ROS, including superoxide anions (O₂^−^), singlet oxygen (^1^O₂), hydrogen peroxide (H₂O₂), and hydroxyl radicals (HO·) (35, 36). Accumulation of these ROS drives oxidative stress, resulting in lipid peroxidation, protein modification, and DNA damage (37). Experimental evidence demonstrates that UV exposure at doses ranging from 480 to 1920 J/m^2^ significantly elevates intracellular ROS levels in murine melanocyte cell lines (38). Among UVR-induced ROS, hydrogen peroxide represents a predominant and biologically relevant species following UV exposure (39). Consistently, studies exposing multiple human melanocyte subtypes to UVR have shown that H₂O₂ production increases in a time- and dose-dependent manner (38).

Excessive UVR-induced ROS disrupt cellular homeostasis and impair normal cellular functions (40, 41). Oxidative stress leads to DNA damage and activation of the p53 signaling pathway, thereby promoting cell cycle arrest and apoptosis (42, 43). The deleterious effects of oxidative stress primarily arise from ROS-mediated damage to proteins, lipids, and DNA. As mitochondria are both a major source and a principal target of ROS, they are particularly susceptible to UV-induced injury (44). In vitiligo, UV exposure dissipates the mitochondrial membrane potential (ΔΨm), reduces ATP production, and induces the release of cytochrome c into the cytoplasm, leading to activation of caspase-dependent apoptotic pathways (45, 46). In addition, UVR induces mitochondrial DNA (mtDNA) deletions and functional impairment, alterations that are especially prominent in photoaged skin (47, 48). Loss of mtDNA, which encodes essential components of the oxidative phosphorylation machinery, compromises cellular energy metabolism, exacerbates ROS accumulation, and promotes cellular senescence, thereby contributing to cutaneous aging.

Although melanocytes are generally considered relatively tolerant to elevated oxidative stress, UVR exposure renders them more susceptible to additional environmental insults. Under basal conditions, no significant difference in cell viability was observed between primary epidermal melanocytes derived from healthy individuals and perilesional melanocytes isolated from patients with vitiligo. However, exposure to 1.0 mM H₂O₂ resulted in a statistically significant reduction in cell viability in vitiligo-derived melanocytes compared with control melanocytes (*p* < 0.01). These findings demonstrate that melanocytes from vitiligo lesions exhibit increased vulnerability to H₂O₂-induced oxidative stress, highlighting an intrinsic defect in redox resilience (49).

UVR elevates intracellular ROS levels in melanocytes, leading to DNA damage and activation of the p53 signaling pathway (50). In vitiligo, increased ROS accumulation in melanocytes has been associated with enhanced lipid peroxidation, reflecting sustained oxidative injury. Concomitantly, melanocytes from patients with vitiligo exhibit heightened susceptibility to oxidative stress due to reduced activities of key antioxidant enzymes, including catalase and glutathione peroxidase (51). Excessive ROS generation, together with insufficient enzymatic and non-enzymatic antioxidant defenses required to maintain redox homeostasis, ultimately culminates in oxidative stress and melanocyte dysfunction (52).

Prolonged or excessive accumulation of ROS in melanocytes induces cellular damage and promotes the generation of self-antigens through multiple mechanisms, including apoptosis, activation of the unfolded protein response (UPR) secondary to endoplasmic reticulum (ER) stress, and the exposure of calreticulin (CRT)-mediated “eat-me” signals that facilitate phagocytic clearances (51, 53). Under these conditions, damaged melanocytes release damage-associated molecular patterns (DAMPs) into the local microenvironment via cellular debris and exosomes, including inducible heat shock protein 70 (HSP70i), high-mobility group box 1 (HMGB1), S100B, and mitochondrial DNA. These DAMPs, often complexed with melanocyte-specific antigens, subsequently activate innate immune responses through pattern recognition receptors (PRRs) (54). In addition, oxidative stress is thought to promote the formation of neoantigens in melanocytes, with ROS facilitating this process through protein oxidation and carbonylation, thereby further amplifying immune activation and melanocyte targeting (55).

High-dose UVR exposure, such as that occurring during sunburn, induces cutaneous injury that may precipitate vitiligo through the Koebner phenomenon. One proposed mechanism underlying this deleterious effect is the impaired capacity of vitiligo melanocytes to adapt to irradiation-induced oxidative stress.

### 3.2 DNA damage and genomic instability

UVR induces extensive DNA damage in cutaneous cells, including keratinocytes, melanocytes, and fibroblasts (56). Both UVA and UVB promote the formation of cyclobutane pyrimidine dimers (CPDs) and 6–4 pyrimidine–pyrimidone photoproducts (6–4PPs) (57), which subsequently give rise to single-strand breaks (SSBs), interstrand crosslinks (ICLs), and nucleotide base modifications (46). In melanocytes, DNA injury may not be restricted to the period of irradiation. A distinctive melanin-dependent “dark CPD” pathway shows that CPDs continue to form for more than 3 h after UVA exposure has ceased through chemiexcitation driven by reactive oxygen and nitrogen species (58). Recent work further indicates that these delayed CPDs may account for up to 50% of the total CPD burden and depend primarily on the presence of pigment rather than active melanin biosynthesis (59). In addition, UVB exposure markedly increases levels of phosphorylated histone H2AX (*γ*-H2AX), a well-established marker of DNA double-strand breaks (DSBs) (60). In contrast, UVA predominantly induces oxidative DNA damage through the generation of ROS, leading to the formation of oxidative lesions such as 8-hydroxy-2′-deoxyguanosine (8-OHdG) (61).

Following UVR exposure, cells activate DNA damage response pathways to repair genotoxic lesions; however, UVR can also impair the efficiency of DNA repair processes. Among the repair mechanisms relevant to UV-induced damage and melanoma development, nucleotide excision repair (NER) plays a central role. NER is a versatile DNA repair pathway responsible for removing a broad spectrum of lesions, including UV-induced cyclobutane pyrimidine dimers (CPDs) and 6–4 pyrimidine–pyrimidone photoproducts (6–4PPs). Notably, a recent study employing flow cytometry-based analysis of UV photoproduct repair across different cell cycle phases demonstrated that the majority of melanoma cell lines exhibit defective NER activity during S phase (62).

### 3.3 Cell death

UVR profoundly affects keratinocyte survival by inducing apoptosis. UVB exposure activates the p53 signaling pathway, promotes mitochondrial depolarization, and drives the accumulation of ROS (45). Excessive ROS increase mitochondrial membrane permeability, leading to cytochrome *c* release into the cytoplasm and initiation of the caspase-dependent apoptotic cascade (46). In parallel, UVB downregulates the antiapoptotic protein Bcl-2, further facilitating apoptotic cell death (63). Enhanced keratinocyte apoptosis following UVB exposure has been confirmed by increased terminal deoxynucleotidyl transferase dUTP nick end labeling (TUNEL) positivity and caspase-3 activation (64). In addition, UV radiation activates mitogen-activated protein kinase (MAPK) and phosphoinositide 3-kinase/protein kinase B (PI3K/AKT) signaling pathways, both of which are critical regulators of cell survival and apoptosis (65). Beyond keratinocytes, UVR also modulates autophagy in dermal fibroblasts in a dose- and time-dependent manner (48). Short-term UVA exposure induces autophagy, facilitating the clearance of damaged cellular components and contributing to cytoprotective responses (66). In contrast, prolonged UVA exposure or high-dose UVB suppresses autophagic flux, thereby impairing cellular repair mechanisms and promoting fibroblast dysfunction and skin aging (67).

UVR induces melanocyte apoptosis through multiple, partially overlapping molecular pathways. UVB directly inflicts genotoxic stress by generating cyclobutane pyrimidine dimers (CPDs), 6–4 pyrimidine–pyrimidone photoproducts (6–4PPs), and DNA strand breaks, thereby activating intrinsic apoptotic signaling cascades (68). In contrast, UVA predominantly acts through indirect mechanisms, inducing the generation of ROS that give rise to oxidative DNA lesions—such as 8-hydroxy-2′-deoxyguanosine (8-OHdG)—as well as DNA strand breaks and protein–DNA crosslinks (36, 41). Intermediates of melanin synthesis further sensitize melanocytes to UVA-induced injury, amplifying ROS-dependent cytotoxicity. Beyond direct genotoxic effects, UVR modulates the expression and activity of apoptosis-related proteins, including p53 and p73, leading to cell-cycle arrest and genomic instability. Concurrently, alterations in the cutaneous immune microenvironment—such as Langerhans cell dysfunction and elevated prostaglandin E₂ (PGE₂) levels—further influence melanocyte apoptotic responses (68). Under specific conditions, UVA exposure also disrupts circadian regulation through suppression of the clock gene *PER1*, which can synergistically enhance apoptotic signaling (69). Collectively, these mechanisms contribute to melanocyte loss in pigmentary disorders such as vitiligo and, under chronic or dysregulated conditions, may also facilitate melanoma development.

### 3.4 Inflammation

UVR, particularly UVB, profoundly modulates the immune–inflammatory microenvironment surrounding melanocytes through multiple mechanisms. UVR directly induces DNA damage in melanocytes and activates the cutaneous neuro–immuno–endocrine system, thereby promoting the release of proinflammatory cytokines, including interleukin-1β (IL-1β), IL-6, and tumor necrosis factor-*α* (TNF-α). These events facilitate immune cell recruitment, enhance ROS production, and collectively trigger localized inflammatory responses (56, 68). Notably, chronically sun-damaged skin exhibits marked overactivation of the IL-17 signaling pathway, which drives neutrophil infiltration via Th17 cells, leading to epidermal barrier disruption and dysregulated melanocyte proliferation (56).

UVR modulates immune function by altering cytokine and chemokine secretion, thereby reshaping the cutaneous immune microenvironment (70). It stimulates the production of antimicrobial peptides (AMPs), which enhance host defense and regulate inflammation (70). UVR also upregulates the immunosuppressive cytokine interleukin-10 (IL-10) while downregulating miR-9 in an IL-10-dependent manner (66), a mechanism that may contribute to immune tolerance and the depigmentation observed in vitiligo (71).

Conversely, UVR exerts robust immunosuppressive effects. Upon UVB exposure, Langerhans cells (LCs) migrate to regional lymph nodes, where they promote the induction of regulatory T cells (Tregs) and secrete immunosuppressive cytokines, including interleukin-10 (IL-10) (72). UV-induced DNA damage acts as a key trigger for LC migration and functional modulation. Mast cells further contribute by releasing mediators with immunoregulatory activity, thereby attenuating cutaneous immune responses (71). Together, these mechanisms establish a paradoxical environment in which inflammation coexists with immunosuppression, facilitating skin aging, compromising host defense, and promoting UV-associated carcinogenesis (73).

## 4 UVR and melanocyte-related diseases

### 4.1 Vitiligo: therapeutic mechanism

UV phototherapy mediates therapeutic effects in diverse dermatological conditions through well-characterized immunomodulatory and anti-inflammatory mechanisms. These effects are orchestrated via multiple interconnected molecular pathways. First, both UVB and PUVA profoundly modulate antigen presentation by impairing epidermal Langerhans cell (LC) function, downregulating co-stimulatory molecules, such as CD80/CD86, and promoting LC migration to lymph nodes (74). This shift preferentially induces regulatory T cells (Tregs) over effector T cells, thereby establishing local immune tolerance (70). Second, phototherapy systematically corrects disease-specific cytokine imbalances. In Th1/Th17-driven psoriasis, it reduces key proinflammatory cytokines, including TNF-α, IL-23, and IL-17 (75), whereas in Th2-dominated atopic dermatitis, it suppresses IL-4 and IL-13 expression (68). Simultaneously, phototherapy enhances the production of anti-inflammatory cytokines such as IL-10 and TGF-*β* across conditions (76), whereas in Th2-dominated atopic dermatitis, it suppresses IL-4 and IL-13 expression (70). Simultaneously, phototherapy enhances the production of anti-inflammatory cytokines such as IL-10 and TGF-β across conditions (77). Additionally, UVB generates tryptophan-derived photometabolites, such as 6-formylindolo[3,2-b]carbazole (FICZ), which act as endogenous ligands for the aryl hydrocarbon receptor (AhR) (78). AhR activation not only regulates melanogenesis but also suppresses proinflammatory cytokine production, including IL-17, while promoting Treg differentiation, contributing critically to the treatment of both psoriasis and atopic dermatitis. Finally, keratinocytes rapidly release platelet-activating factor (PAF) upon UV exposure. PAF binding to its receptor triggers a signaling cascade leading to the release of mediators such as prostaglandin E2 (PGE2), a pathway central to systemic immunosuppression (79). Genetic deletion or pharmacological inhibition of the PAF receptor effectively abrogates UV-induced immunosuppression (79).

*In vivo* evidence from both animal models and human studies consistently demonstrates that UV, particularly UVB, enhances melanogenesis and promotes repigmentation in vitiligo. In murine and guinea pig models, UVB exposure induces visible skin darkening, accompanied by a pronounced increase in melanin deposition within the basal epidermal layer, as confirmed by histological analyses such as Fontana–Masson staining (12). These pigmentation changes reflect the coordinated activation of melanogenic programs at the tissue level, resulting in increased melanin synthesis in intact skin.

In humans, repeated UV or narrowband UVB (NB-UVB) exposure elicits robust pigmentary responses that closely mimic physiological tanning and therapeutic repigmentation in vitiligo. Clinical studies of NB-UVB therapy report substantial repigmentation after prolonged treatment, with non-acral lesions responding more favorably than acral sites (20). The predominant repigmentation patterns, such as perifollicular pigment islands and marginal pigment spread, support a follicular origin of melanocyte repopulation *in vivo* (80). *Ex vivo* human skin and pigmented skin equivalent models further confirm that UVB exposure increases overall melanin content and basal-layer melanin accumulation, paralleling changes observed in sun-exposed human skin (20). Collectively, these findings indicate that UV-induced melanogenesis in vitiligo is a coordinated tissue-level process, reliant on the integrity of the cutaneous microenvironment rather than isolated cellular responses.

UV-based therapies exert potent immunomodulatory effects *in vivo*, contributing to disease stabilization in vitiligo. Clinical studies consistently show that narrowband UVB (NB-UVB) treatment markedly reduces disease activity in patients with active vitiligo, as evidenced by significant decreases in VIDA scores following therapy (20). Patients in an active disease phase at baseline exhibited substantial suppression of ongoing lesion activity after NB-UVB exposure, indicating attenuation of immune-driven disease progression. These findings support the notion that UV irradiation mitigates pathogenic immune responses against melanocytes in human skin.

At the tissue level, UV exposure modulates the local immune microenvironment by reshaping cytokine and chemokine networks within the skin. UV irradiation can alter the release of IFN-*γ*-associated chemokines, such as CXCL9 and CXCL10, thereby limiting the recruitment and persistence of cytotoxic T cells in lesional skin (80). In animal models, UVB exposure induces epidermal thickening and immune cell infiltration, reflecting an acute inflammatory response capable of remodeling local immune dynamics (68). Keratinocyte-derived mediators, such as chemokines and growth factors, further regulate immune cell trafficking and survival within UV-exposed skin, reinforcing these local immunomodulatory effects.

In clinical practice, the immunomodulatory effects of UV therapy are often potentiated by combination treatment with topical tacrolimus, which suppresses T cell activation and diminishes cytotoxic immune responses against melanocytes (81). Emerging clinical evidence also suggests that NB-UVB therapy may indirectly modulate systemic immunity by improving sleep quality and restoring circadian melatonin rhythms, both of which exhibit anti-inflammatory properties (29). Collectively, data from human and animal studies indicate that UV irradiation regulates local and systemic immune responses, thereby attenuating autoimmune activity and promoting disease stabilization in vitiligo.

### 4.2 Melanoma: carcinogenic mechanisms

Although melanin induced by UV radiation (UVR) provides antioxidant protection, it also renders melanocytes more vulnerable to damage. Excessive UVR or environmental stressors increase melanocyte susceptibility. *In vitro* studies show that exposing human epidermal melanocytes to two doses of 20 mJ/cm^2^ UVB over 24 h induces cellular senescence and pigmentation (82). Similarly, the skin-whitening agent 4-(4-hydroxyphenyl)-2-butanol (Rhododendrol, RD) has been reported to cause depigmentation in some users due to its cytotoxicity toward melanocytes, an effect exacerbated by concurrent UVR exposure (83). UVR, particularly UVB, is a well-established environmental carcinogen and a major risk factor for melanoma. Population-based analyses using national and subnational cancer registries indicate that the majority of melanomas worldwide in 2022 were attributable to UV exposure, based on data from 154 countries (84). UV directly damages DNA by generating cyclobutane pyrimidine dimers (CPDs) and 6–4 pyrimidine–pyrimidone photoproducts (6–4PPs), which serve as initiating lesions in skin carcinogenesis. UV-induced mutations frequently localize to specific loci, such as the p. R1393Q variant of ZNF831, disrupting melanocyte proliferation, apoptosis, and migration, and potentially compromising tumor-suppressive functions to promote melanoma development (85). Experimental studies further support this link: neonatal mice exposed to UV developed melanoma in 50% of cases by 12 weeks and in 100% by 24 weeks (38), and loss of p16 accelerates UV-induced melanoma formation. Endogenous melanin provides partial protection by absorbing UV photons and ROS; however, excessive UV exposure can oxidize melanin, generating ROS. Notably, even in the absence of UV, melanin biosynthesis is intrinsically pro-oxidative, correlating with higher basal intracellular ROS levels in melanocytes, which are further elevated upon p16 depletion. Thus, melanin functions as a double-edged sword: it shields melanocytes and neighboring keratinocytes from UV-induced damage, yet its synthesis increases intracellular ROS, potentially heightening melanoma susceptibility. Further studies are needed to determine whether this pro-oxidative property of melanin underlies the increased melanoma risk observed in individuals with inherited p16 mutations, relative to other cancers (86–88).

## 5 Clinical implications and safety considerations

### 5.1 Phototherapy modalities

UVR can induce repigmentation of depigmented lesions and represents a cornerstone therapy for vitiligo. Phototherapy utilizes specific UV wavelengths, such as UVA, psoralen–UVA (PUVA), and UVB. PUVA therapy involves systemic or topical administration of a photosensitizer, followed by UVA exposure. Under UVA irradiation, the photosensitizer forms photochemical adducts with thymine in DNA, thereby inhibiting DNA replication and suppressing cell proliferation and inflammation (89). UVB-based therapies include broadband UVB (BB-UVB, 280–320 nm) and narrowband UVB (NB-UVB, 311–313 nm), with NB-UVB currently considered the clinical standard for vitiligo treatment (90) (Table 2).

**Table 2:** Classification and mechanism of UV light therapy.

Phototherapy modality	Mechanism/therapeutic effects
PUVA	Increase the quantity and activity of epidermal melanocytes, while reducing the degenerative changes of melanocytes and corneal forming cells, in order to combat further pigment loss.
NB-UVB	Downregulated inflammatory cytokines and upregulated interleukin(IL-10) to inhibit local T lymphocytes and promote Th17/Tregs balance Promotes the proliferation, migration, and differentiation of melanocytes in hair follicles and lesional skin, thereby contributing to both disease stabilization and repigmentation Increase the expression of Tyrosinase protein (Tyr), Tyrosinase-related protein 1 (Tyrp1), and Tyrosinase-related protein 2 (Tyrp2) genes to stimulate melanin synthesis (114) Elevate the level of 25-hydroxyvitamin D, as vitamin D is involved in melanin deposition Upregulate the expression of Wnt7 in the epidermis and promote the translocation of β-catenin to melanocyte stem cells (McSCs) (99, 115) stimulate basic fibroblast growth factor (bFGF) and endothelin-1 (ET-1) releasing from keratinocytes induces melanocyte proliferation (116, 117)
308nmUVB	Induce pathological T lymphocyte apoptosis in the lesion (118) By influencing the vitality of keratinocytes, keratinocytes secrete stem cell factor (SCF), granulocyte-macrophage colony-stimulating factor (GM-CSF), basic fibroblast growth factor (bFGF), and other cytokines that promote the proliferation, differentiation, and synthesis of melanocytes, and indirectly act on melanocytes through the above cytokines. Induce melanocyte migration, proliferation, differentiation, and accelerate melanocyte synthesis. Through its action on keratinocytes to indirectly induce T-cell apoptosis, transforming growth factor-β1 (TGF-β1) plays a crucial role in the apoptosis of T lymphocytes induced by excimer laser. Enhance the retention of residual melanocytes in the outer root sheath of the peripheral hair follicle (119)

### 5.2 Advances in combination phototherapy with NB-UVB for vitiligo

**Figure 2:**
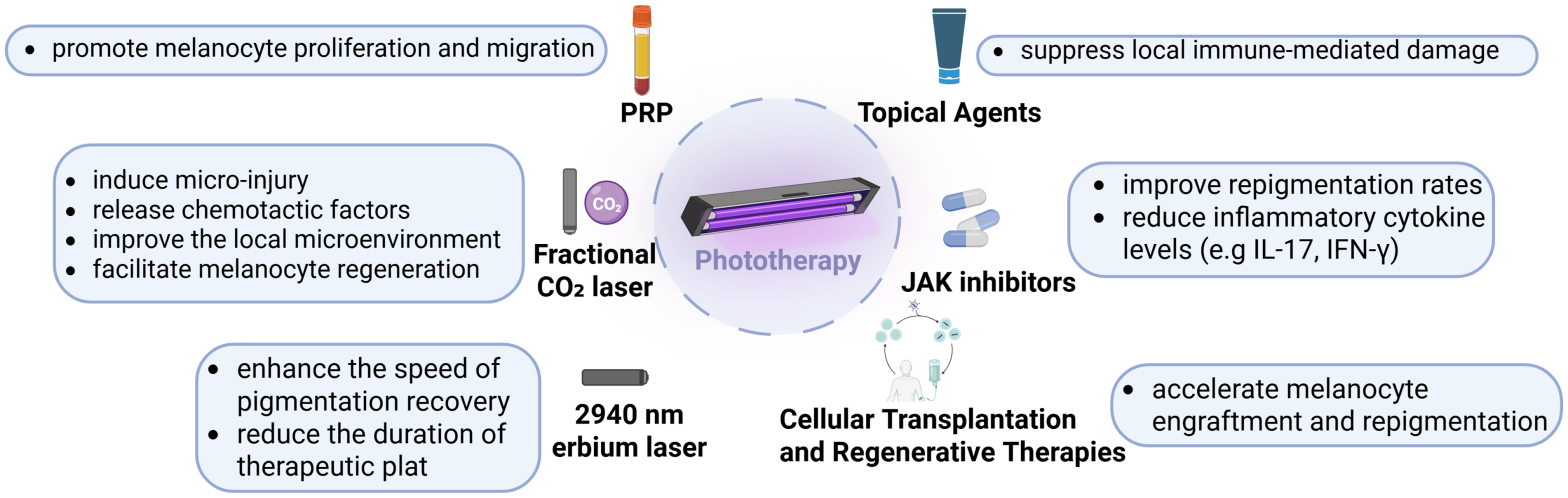
Combination phototherapy for vitiligo (created at https://BioRender.com).

Phototherapy, particularly narrowband UVB (NB-UVB) and 308-nm excimer lasers, has become a cornerstone of vitiligo treatment (91). In recent years, combination therapies integrating phototherapy with other modalities have demonstrated promising synergistic effects. Platelet-rich plasma (PRP), rich in growth factors, promotes melanocyte proliferation and migration. While some studies report enhanced repigmentation with NB-UVB plus PRP, meta-analyses reveal no significant advantage, highlighting the need for high-quality randomized controlled trials (92). Fractional CO₂ lasers induce microinjury and release chemotactic factors, facilitating melanocyte regeneration. Triple therapy (NB-UVB + PRP + laser) improves clinical responses and reduces non-responders (93). The 308-nm excimer laser combined with PRP enhances repigmentation, whereas the 2,940-nm erbium laser accelerates pigmentation recovery in treatment-resistant vitiligo (94). NB-UVB, combined with calcineurin inhibitors (e.g., tacrolimus), yields superior outcomes in facial and cervical vitiligo by promoting melanocyte activity and suppressing immune-mediated damage (13). Excimer lasers combined with corticosteroids, vitamin D analogs, or antioxidants also show synergistic effects (95). Among emerging strategies, JAK inhibitors are especially noteworthy because they directly target IFN-*γ*-driven JAK–STAT signaling and the downstream CXCL9/CXCL10 axis, thereby reducing cytotoxic T-cell recruitment and persistence in lesional skin (96–98). When paired with NB-UVB, this immune blockade appears mechanistically complementary to phototherapy-induced melanocyte activation: NB-UVB promotes melanocyte stem-cell differentiation and migration through Wnt/*β*-catenin and Kit signaling and enhances keratinocyte-derived melanogenic mediators, such as endothelin-1 and bFGF (99). A recent systematic review and meta-analysis of four controlled studies, including 217 adults, found that JAK inhibitor plus NB-UVB significantly improved repigmentation versus NB-UVB alone, including a 6.87-fold higher probability of achieving ≥50% repigmentation and a 15.13-fold higher probability of achieving ≥75% repigmentation (96). These pooled findings are supported by recent clinical studies. In a randomized controlled trial, tofacitinib plus NB-UVB improved overall clinical effectiveness, VASI, DLQI, and inflammatory cytokine profiles compared with NB-UVB monotherapy (97). Likewise, both a phase 2 randomized clinical trial and a prospective controlled study showed that baricitinib combined with NB-UVB reduced disease activity and produced faster, clinically meaningful repigmentation without major additional safety signals (74). NB-UVB with non-cultured epidermal or follicular cell grafts accelerates repigmentation, while microneedle-assisted PRP improves color matching (95). In summary, combination phototherapy enhances repigmentation by promoting melanocyte regeneration, modulating immune responses, and improving patient adherence. However, current evidence remains limited by modest sample sizes, heterogeneous designs, and relatively short follow-up, underscoring the need for robust multicenter trials with standardized outcome measures (96–98) (Figure 2).

### 5.3 Phototherapy in vitiligo: evaluation of safety and efficacy

#### 5.3.1 Efficacy

Narrowband UVB (NB-UVB), the 308-nm excimer laser, and excimer lamps demonstrate comparable efficacy, with excimer devices often inducing earlier repigmentation in facial and neck lesions. Clinical trials report that approximately 40–60% of treated lesions achieve ≥75% repigmentation within 9–12 months (100). Combination therapies with topical tacrolimus or JAK inhibitors further improve outcomes, with randomized controlled trials showing ≥75% repigmentation in over 65% of patients at 24 weeks (101). Regenerative approaches, such as melanocyte–keratinocyte transplantation (MKTP) and PRP, provide additional benefit (102). Improvements in the Vitiligo Area Scoring Index (VASI) and Dermatology Life Quality Index (DLQI) corroborate these clinical gains, although acral sites remain relatively resistant to treatment (103).

#### 5.3.2 Safety

Phototherapy is generally well-tolerated. Adverse effects, such as erythema, pruritus, and transient hyperpigmentation, are typically mild and self-limiting, with severe reactions being rare (1, 2). Importantly, no convincing signal of increased skin cancer has been established with long-term NB-UVB therapy in vitiligo cohorts to date (104). However, the epidemiologic interpretation of melanoma risk in vitiligo is more nuanced than a simple cumulative-UV model. A 2025 Swedish population-based matched cohort study including 15,030 patients with vitiligo reported a lower risk of malignant melanoma than in the general population (HR 0.53, 95% CI 0.32–0.86) (105). This apparent melanoma paradox may reflect a combination of heightened immune surveillance against melanocytic antigens, disease-associated genetic architecture, and altered UV-exposure behavior, although the relative contribution of these mechanisms remains unresolved (105). Combination treatment with JAK inhibitors does not result in major additional toxicity, although mild pruritus or photosensitivity may occur (75). Home-based NB-UVB therapy is also safe, with grade 3–4 adverse reactions reported in fewer than 10% of treatments (103). Overall, these data are reassuring but should not eliminate the need for individualized photoprotection and long-term surveillance.

Mechanistically, UVB suppresses Dicer expression via the PI3K/RSK/Wnt–β-catenin pathway, disrupting microRNA biogenesis and impairing melanocyte function (106). Additionally, UVB modulates melanocyte stem cell migration through TNF signaling, providing a potential mechanistic link between chronic UV exposure and melanoma initiation (107).

#### 5.3.3 Clinical strategies in phototherapy for vitiligo

Treatment protocols typically employ individualized dosing of narrowband UVB (NB-UVB) or 308-nm excimer laser therapy, often starting at the minimal erythema dose (MED) or approximately 100 mJ/cm^2^, administered two to three times per week for 12–36 weeks (103, 108). Efficacy is assessed using the Vitiligo Area Scoring Index (VASI), Dermatology Life Quality Index (DLQI), repigmentation rates, and patient-reported outcomes, while reflectance confocal microscopy (RCM) allows microscopic evaluation of melanocyte regeneration (109). Emerging strategies combine phototherapy with JAK inhibitors or topical agents, such as bimatoprost, tacrolimus, or calcipotriol, demonstrating synergistic effects, particularly when paired with natural light exposure in patients receiving systemic immunomodulators (101).

## 6 Conclusion

UVR exerts bidirectional effects in vitiligo: it can act as a pathogenic factor by inducing oxidative stress, DNA damage, apoptosis, and immune dysregulation, yet also functions therapeutically by promoting melanocyte proliferation, migration, and differentiation via Wnt/*β*-catenin activation and immune modulation, including regulatory T cell induction and Th17 suppression (110, 111). In addition, melanocytes should be considered UV-responsive regulatory cells rather than merely pigment-producing cells, and the balance between eumelanin and pheomelanin may critically influence whether UV-induced melanogenesis results in photoprotection or oxidative injury. Although NB-UVB remains the gold standard for vitiligo treatment, UVR-induced oxidative stress may impair skin barrier function and increase melanocyte vulnerability. Phototherapy is well-tolerated, and currently, no clinical evidence suggests an increased risk of cancer with NB-UVB treatment for vitiligo, and long-term monitoring for photocarcinogenesis remains prudent (81, 112, 113). Combination strategies incorporating PRP, JAK inhibitors, or cellular grafts further enhance clinical outcomes. Future research should focus on optimizing phototherapy protocols, elucidating melanocyte stem-cell dynamics, and developing targeted interventions that balance UVR’s pathogenic and therapeutic actions, integrating advances in photobiology, immunology, and regenerative medicine to improve vitiligo management (107).

## Glossary

6–4PPs: 6–4 pyrimidine-pyrimidone photoproducts
8-OHdG: 8-hydroxy-2′-deoxyguanosine
AMPs: antimicrobial peptides
bFGF: basic fibroblast growth factor
cAMP: the cyclic adenosine monophosphate
cGMP: cyclic guanosine monophosphate
CHS: contact hypersensitivity
CPDs: cyclobutane pyrimidine dimers
CRH: Corticotropin-releasing hormone
Cyt C: cytochrome c
DCT: dopachrome tautomerase
DLQI: patient quality of life
DSBs: DNA double-strand breaks
ET-1: endothelin-1
ICLs: interstrand crosslinks
IL-10: interleukin-10
IL-1β: interleukin-1β
JAK: Janus Kinase
MAPK: mitogen-activated protein kinase
MaR1: maresin 1
MC1R: melanocortin 1 receptor
MC1R: melanocortin-1 receptor
McSCs: melanocyte stem cells
MED: minimal erythema dose
miRNAs: microRNAs
mtDNA: mitochondrial DNA
NB-UVB: narrowband ultraviolet B
NF-κB: nuclear factor kappa B
Opn4KO: OPN4-knockout
Opn4WT: OPN4 wild-type
PI3K/AKT: phosphoinositide 3-kinase/protein kinase B
POMC: pro-opiomelanocortin
PRP: Platelet-Rich Plasma
RCTs: randomized controlled trials
ROS: oxidative stress
RvD1: Resolvin D1
SASP: senescence-associated secretory phenotype
SPF: sun protection factor
SSBs: single-strand breaks
TNF-*α*: tumor necrosis factor-α
Tregs: regulatory T cells
UV: ultraviolet
UVA: ultraviolet A
UVB: ultraviolet B
UVR: Ultraviolet radiation
VASI: Vitiligo Area Scoring Index
α-MSH: α-melanocyte-stimulating hormone
β2-AR: β2-adrenergic receptors
*γ*-H2AX: phosphorylated histone H2AX
ΔΨm: The mitochondrial membrane potential
